# Knowledge, beliefs and practices regarding prevention of bacterial meningitis in Burkina Faso, 5 years after MenAfriVac mass campaigns

**DOI:** 10.1371/journal.pone.0253263

**Published:** 2021-07-14

**Authors:** Judith E. Mueller, Joy Seanehia, Seydou Yaro, Caroline L. Trotter, Ray Borrow, Tamara Giles-Vernick

**Affiliations:** 1 EHESP French School of Public Health, Rennes and Paris, France; 2 Institut Pasteur, Paris, France; 3 Centre Muraz, Bobo-Dioulasso, Burkina Faso; 4 University of Cambridge, Cambridge, United Kingdom; 5 Vaccine Evaluation Unit, Public Health England, Manchester, United Kingdom; Uniwersytet Zielonogorski, POLAND

## Abstract

**Background:**

To adapt communications concerning vaccine prevention, we studied knowledge, beliefs and practices around meningitis risk and prevention in a young adult population in Burkina Faso in 2016, 5 years after the MenAfriVac® mass campaign and one year before the vaccine’s inclusion in the infant immunization schedule.

**Methods:**

In a representative sample of the population aged 15 to 33 years (N = 220) in Bobo-Dioulasso, Burkina Faso, study nurses administered a standardized paper questionnaire consisting of predominantly open questions, collecting information on meningitis risk factors and prevention, and on exposure to dry air and kitchen fire smoke. We identified themes and analyzed their frequency. We created a meningitis knowledge score (range 0 to 4) based on pre-defined best responses and analyzed the determinants of knowledge score levels ≥2 (basic score) and ≥3 (high score) using multivariate logistic regression.

**Results:**

Biomedically supported facts and good practices were known by the majority of participants (eg vaccine prevention, 84.5%). Younger women aged 15–20 years had a higher frequency of low scores <2 (17.0%) compared to older women aged 21–33 years (6.3%) and men of both age groups (3.8%). Junior secondary School attendance explained the differences between the two groups of women, the gender gap for the older, but not the young women, and explained score differences among young women. Local understandings and practices for risk and prevention were commonly reported and used (risk from unripe mango consumption and prevention through nasal application of shea nut butter).

**Discussion:**

This study shows a gender gap in knowledge of meningitis risk and prevention, largely due to education-level inequalities. Women below 21 years had particularly low levels of knowledge and may need interventions outside schools and perinatal care. Our study suggests a strong adherence to local understandings of and practices around meningitis risk and prevention, which should be taken into account by vaccination promotion.

## Introduction

The African meningitis belt, which spans the sub Saharan African Sahel region from Senegal in the west to Ethiopia in the east, is characterized by a high incidence of bacterial meningitis. A long dry season combines with a meteorological phenomenon in which the Harmattan wind from the northeast brings dry air with high mineral aerosol load. As a consequence, African populations inhabiting the meningitis belt are exposed to conditions of low relative humidity and dust clouds of large and fine mineral particles [1]. These conditions, occurring between December and April each year, are related to a hyperendemic incidence of meningococcal and pneumococcal meningitis [2]. Although no biomedical mechanism is confirmed, it is assumed that the noxious effects of dry dusty air on the nasopharyngeal mucosa facilitates the invasion of bacteria (and possibly transmission), which naturally colonize the nasopharynx [3, 4]. In addition, sporadic localized epidemics of meningococcal meningitis occur and micro-epidemics of respiratory virus infectious have been suggested as a co-factor [5]. Larger epidemic waves occur every 7 to 10 years, probably in relation to the emergence of new meningococcal strains [4]. The highest incidence rates are observed among young children, but adolescents and young adults are also at a high risk of both meningococcal and pneumococcal meningitis [6]. Specific individual risk factors that have been identified during localized epidemics are exposure to kitchen fire smoke [7, 8] and recent respiratory infection [8].

Several vaccines against meningitis are used in the belt populations, including *Haemophilus influenzae* type b conjugate vaccine and 13-valent pneumococcal conjugate vaccine in the Expanded Programme on Immunization (EPI). In 2010, a serogroup A conjugate vaccine (MenAfriVac®) was introduced through mass campaigns among persons aged 1–29 years with the aim of interrupting meningococcal circulation in the population and providing individual immune protection for several years [9]. From 2016 onwards, the vaccine has been introduced in the EPI to protect new birth cohorts. Since these efforts were introduced, serogroup A meningococcal meningitis has disappeared from most meningitis belt countries, while a high incidence of pneumococcal meningitis persists among older children and adults. The remaining epidemic events and hyperendemic cases are due to serogroups C, W, and X, which are or will be covered by other vaccines [10, 11].

The MenAfriVac® mass campaigns were accompanied by a large mobilization of civil society and communication efforts. The experience of recurrent meningitis epidemics with high attack rates (up to 1% of the population affected during an epidemic), high case fatality (10% for meningococcal meningitis and 30% for pneumococcal meningitis) and substantial economic burden due to treatment and frequent long-term sequelae [12] lead to high acceptance of vaccination and high coverage during mass campaigns [9].

Few scientific publications have described the populations’ beliefs with regard to meningitis and its vaccine prevention. A qualitative study in different towns in Burkina Faso before the MenAfriVac® campaign described the frequent parallel consultation of modern and traditional medicine and spiritual practice for meningitis treatment [12]. A case-control study in Ghana evaluated knowledge about symptoms and health care-seeking behavior and described knowledge about treatment of the disease [13]. About half of the participants reported crowded sleeping and lack of ventilation and 80% reported heat as a risk factors for meningitis.33% mentioned vaccination for prevention [13]. To adapt communications concerning vaccine-induced prevention to the population’s information needs and beliefs, for example by mobilizing local knowledge for vaccine promotion, we studied knowledge, perceptions and practices around meningitis risk and prevention in a young adult population of Burkina Faso in 2016, 5 years after the MenAfriVac® mass campaign that targeted the 1–29 year-old population and one year before the vaccine’s inclusion in the infant immunization schedule.

## Materials and methods

### Data collection

As described in detail elsewhere, we conducted a series of three seroprevalence surveys (2011, 2013 and 2016) in Bobo-Dioulasso, the second largest town of Burkina Faso [14]. During the third survey conducted from January through February 2016, we included a questionnaire to evaluate knowledge, beliefs and practice of meningitis prevention. We established a representative sample of the population aged 6 months to 32 years based on a two-stage cluster sampling design, selecting cross roads and compounds. Participants were randomly selected from compound members in the required age group. Written consent was sought from participants or the legal guardians of <18 year olds and where participant was illiterate, a fingerprint was obtained in the presence of an independent witness. Included participants were invited to a study visit at the Centre Muraz a few days later. The present analysis carried on all interviews conducted with ≥15-year-old participants of the survey. At the study center, a standardized paper questionnaire consisting of predominantly open questions was administered by study nurses to collect data on knowledge, beliefs and perception of meningitis risk factors and prevention, and on exposure to dry air and kitchen fire smoke (**S1 Table**). The number of responses were not limited and participants could give more than one response per question. We designed this questionnaire based on a pilot questionnaire administered during the second seroprevalence survey in 2013 and anthropological and public health expert knowledge. The questionnaire was developed and filled out in French. During training of the field workers and study nurses, a translation to the two most prevalent local languages (Dioula and Moré) was defined by the field team and used during the study interviews. Answers were written in a paper questionnaire and entered into a database by one data manager. Study nurses were consulted during entry in case of unclear interpretation of wording. Institutional Review Board approval was obtained from National Ethics and Health Research Committee of Burkina Faso, and the Institutional Ethics Committee of Centre Muraz, Bobo-Dioulasso, Burkina Faso.

### Data analysis and interpretation

In the present analysis, only participants aged 15 years old or older were included. Regarding beliefs and practices, we identified themes and assigned keywords, based on the free-text responses provided to open questions. During this process, all responses to a given question were listed and relevant themes identified (eg, “application of shea butter” and “covering one’s nose” as preventative practices). We created variables for the mention of each theme (modalities: yes vs. no), which then allowed conducting statistics on the frequency of the theme.

We conducted descriptive analyses on the frequency of different themes, stratifying by age (15–20 years vs. 21–33 years) and gender. Two-sided Fisher’s exact test was used to assess the significance of differences.

With regards to meningitis pathophysiology, risk factors and prevention identified in epidemiological studies, we created a meningitis knowledge score using one pre-defined biomedical best response per question (**S1 Text**): persons at highest risk of meningitis: children (and “young people”); the effect of dry air on health: meningitis; mode of infection with meningitis: (droplets from) person to person; prevention: vaccination. We allocated one point for each correct response, thus the knowledge score ranged from 0 to 4. We analyzed the association of knowledge score levels ≥2 (basic score) and ≥3 (high score) with participant characteristics (education and proxies of socioeconomic status) using bivariate and multivariate logistic regression. Variables contributing at *P*<0.20 in bivariate analyses were included in a multivariate model and stepwise exclusion used based on their *P*-value, leaving only variables contributing at P<0.05. The overall contribution of variables with more than two modalities was assessed using the Wald test. Based on the hypothesis that gender and age (≥/< 21 years) interact in their effects on knowledge, we built an additional mode including a combined variable distinguishing younger women, younger men, older women and older men (M1). We then evaluated whether at least one year of junior secondary school explained the observed association between knowledge, age and gender (M2). In a third model (M3), we evaluated specifically among young women (the group with the lowest knowledge scores), whether junior secondary school education and satisfaction with own information level were associated with knowledge score. Odds ratios and exact *P*-values are shown. All analyses were carried out using STATA software version 14. Data is available as supporting material.

## Results

### Study population

Complete information was available for 200 of the 223 participants aged 15 years or older. Among them, age ranged from 15.0 to 33.4 years (mean 22.5 years, SD = 4.8). There were 47 women (21.4% of total sample) and 39 men (17.7%) aged 15–20 years, and 96 women (43.6%) and 38 men (17.3%) aged 21–33 years (**Table 1**). Recall-based and document-confirmed MenAfriVac® vaccination during the 2010 mass campaign (all participants were eligible) was found for 64.6% and 13.2% of participants, respectively. About half of participants (46.4%) were students, 72.4% declared being able to read a text in French language, and 64.6% had attended at last one year of junior secondary school (“collège”). Educational indicators showed a large gender disparity: 20.5% and 33.7%, respectively, of 15-to 20-year-old and 21-to 33-year-old women reported being illiterate, vs. 2.6% and 8.3%, respectively, among the corresponding male age groups (*P*-values = 0.033 and 0.004). Attendance of at least one year of junior secondary school was reported for both female age categories as 66.0% and 46.9% respectively, vs. 84.6% and 86.8%, respectively, for men (**Table 1**). A gender gap was also observed for ≥1h of daily exposure to kitchen fire smoke, which was 40.4% and 33.3% among women, vs. 5.1% and 7.9% among men. By contrast, the percentage sharing dinner with >10 persons (overall 32.9%) and living in a household with an open air kitchen or one located in a hangar (52.3%) was similar among gender groups. The most commonly cited fuel for cooking was charcoal (40.9%), followed by firewood (24.1%) or combinations of firewood with other fuels (18.0%). Only 8.6% of households used gas exclusively. All households reported having a radio.

**Table 1:** Characteristics of study participants by age and gender among 220 young adults in Bobo-Dioulasso, Burkina Faso, 2016.

Characteristics	Total	Women 15–20 yrs	Men 15–20 yrs	Women 21–33 yrs	Men 21–33 yrs
	N = 220	N = 47	N = 39	N = 96	N = 38
Mean age in years (standard deviation)	22.5 (4.8)	17.6 (1.5)	18.0 (1.3)	25.8 (3.6)	25.1 (3.8)
**Vaccinated with MenAfriVac**					
Document-confirmed	29 (13.2)	3 (6.4)	5 (12.8)	16 (16.7)	5 (13.2)
Recall	142 (64.6)	25 (53.2)	30 (76.9)	64 (66.7)	23 (60.5)
**Professional activity**					
Student	102 (46.4)	26 (55.3)	34 (87.2)	22 (22.9)	20 (52.6)
Housewife, housekeeper	51 (23.2)	13 (27.7)	0	34 (35.4)	4 (10.5)
Vendor	35 (15.9)	3 (6.4)	1 (2.6)	28 (29.2)	3 (7.9)
Artisan	24 (10.9)	4 (8.5)	4 (10.3)	10 (10.4)	6 (15.8)
Employee	7 (3.2)	1 (2.1)	0	2 (2.1)	4 (10.5)
Civil servant	1 (0.5)	0	0	0	1 (2.6)
**Can you read a newspaper or a letter written in French**					
No	44 (21.0)	9 (20.5)	1 (2.6)	31 (33.7)	3 (8.3)
A little	14 (6.7)	4 (9.1)	2 (5.3)	7 (7.6)	1 (2.8)
Yes	152 (72.4)	31 (70.5)	35 (92.1)	54 (58.7)	32 (88.9)
Highest level of education *					
No schooling or Lower primary (*CP*)	30 (13.6)	10 (21.3)	0	20 (20.8)	0
Upper primary (*CM2*)	48 (21.8)	6 (12.8)	6 (15.4)	31 (32.3)	5 (13.2)
Junior secondary school (*collège*)	87 (39.6)	24 (51.1)	23 (59.0)	26 (27.1)	14 (36.8)
Senior Secondary school (*Lycée et Bac+*)	55 (25.0)	7 (14.9)	10 (25.6)	19 (19.8)	19 (50.0)
**Number of people sharing dinner**					
1–6	91 (41.6)	17 (37.0)	12 (30.8)	43 (44.8)	19 (50.0)
7–9	56 (25.6)	10 (21.7)	14 (35.9)	22 (22.9)	10 (26.3)
10–47	72 (32.9)	19 (41.3)	16 (33.3)	31 (32.3)	9 (23.7)
**Location of kitchen**					
Enclosed	105 (47.7)	20 (42.6)	17 (43.6)	48 (50.0)	20 (52.6)
Hangar	12 (5.5)	5 (10.6)	3 (7.7)	2 (2.1)	2 (5.3)
Open air	103 (46.8)	22 (46.8)	19 (48.7)	46 (47.9)	16 (42.1)
**≥1h of kitchen fire smoke exposure**	56 (25.5)	19 (40.4)	2 (5.1)	32 (33.3)	3 (7.9)
**Most often used fuel**					
Gas	19 (8.6)				
Firewood	53 (24.1)				
Firewood and gas	3 (1.4)				
Firewood, charcoal and gas	9 (4.1)				
Charcoal and gas	19 (8.6)				
Firewood and charcoal	27 (12.3)				
Charcoal	90 (40.9)				

* at least one year in this school level.

The variable “read a newspaper” was available for 210 participants only.

### Meningitis risk and prevention

Half (45.7%) of participants thought that information concerning meningitis risk and prevention was sufficiently accessible, but this opinion was less frequent among younger women (36.2%). The media (TV, radio), health care professionals and school teachers were mentioned most frequently as sources. In turn, health care professionals, the government, TV and radio were most frequently mentioned as sources that should provide information by those who considered that they did not receive enough. Younger women primarily expressed that health care professionals were in charge of providing this information.

Frequently cited effects of dry air were meningitis (72.3%) and lower and upper respiratory infections (76.4% and 68.2%, respectively) (**S2 Table**). Half of participants reported that eating unripe mangos (49.3%) and dust (48.0%) were responsible for meningitis, according to the people in their circle. Only 5, (0.9%), mentioned person-to-person transmission. When asked about their own opinion on what caused meningitis, 58.7% mentioned dust, and 11.9% dust in the respiratory tract (**Table 2**). Among those citing dust, it came from wind (74.2%) or was inhaled (22.6%). Meningitis was associated with fire smoke for 1.9%, along with traffic pollution (1.3%) or unhygienic conditions (2.6%). Furthermore, 29.4% of participants thought themselves that meningitis was caused by eating unripe mangos and 2.8% mentioned contact between persons. Most participants gave only one factor, but a common combination was dust and eating unripe mangos. Younger women less frequently mentioned dust and more frequently did not know any causes.

**Table 2:** Meningitis risk and prevention knowledge, beliefs and practice among 220 young adults by age and gender in Bobo-Dioulasso, Burkina Faso, 2016.

	Total		Women 15–20 yrs		Men 15–20 yrs		Women 21–33 yrs		Men 21–33 yrs	
	N = 220	%	N = 47	%	N = 39	%	N = 96	%	N = 38	%
**Do you think you have enough information on how to protect yourself from meningitis?**										
Yes			17	36.2	18	46.2	48	50.0	17	44.7
**In your opinion, which are the health effects of dry air?**										
LRTI	168	76.4	38	80.9	30	76.9	71	74.0	29	76.3
meningitis	159	72.3	26	55.3	32	82.1	72	75.0	29	76.3
URTI	150	68.2	35	74.5	19	48.7	71	74.0	25	65.8
malaria	21	9.5	3	6.4	3	7.7	9	9.4	6	15.8
other infections*	41	18.6	7	14.9	9	23.1	13	13.5	12	31.6
does not know	1	0.5	1	2.1	0		0		0	
**According to you, how does one get meningitis?**										
							N = 94			
dust	128	58.7	17	36.2	26	66.7	56	59.6	29	76.3
unripe mangos/fruits	64	29.4	16	34.0	10	25.6	30	31.9	8	21.1
dust or smoke in respiratory tract	26	11.9	5	10.6	4	10.3	11	11.7	6	15.8
food hygiene, dirt	12	5.5	5	10.6	2	5.1	4	4.3	1	2.6
person-to-person	6	2.8	2	4.3	2	6.0	2	2.1	0	
wind	3	1.4	1	2.1	0		2	2.1	0	
sun	3	1.4	0		0		3	3.2	0	
does not know	18	8.3	7	14.9	1	2.6	9	9.6	1	2.6
**How can one protect from meningitis?**										
vaccination	186	84.5	39	83.0	34	87.2	78	81.3	35	92.1
avoid dust	32	14.5	5	10.6	4	10.3	18	18.8	5	13.2
unspecific professional help *	17	7.7	3	6.4	1	2.6	8	8.3	5	13.2
does not know / nothing	8	3.6	3	6.4	2	5.1	3	3.1	0	
not eat unripe mangos/fruits	5	2.3	2	4.3	0	0	2	2.1	1	2.6
food hygiene	4	1.8	0		1	2.6	3	3.1	0	
shea butter in the nostrils	3	1.4	0		0		2	2.1	1	2.6
avoid smoke	1	0.5	0		0		1	1.0	0	
mosquito/malaria	1	0.5	0		0		1	1.0	0	
**How do you protect yourself from meningitis?**										
shea butter in the nostrils	101	45.9	15	31.9	23	59.0	49	51.0	14	36.8
vaccination	57	25.9	10	21.3	7	17.9	29	30.2	11	28.9
avoid dust	57	25.9	5	10.6	12	30.8	21	21.9	19	50.0
food hygiene	31	14.1	11	23.4	4	10.3	10	10.4	6	15.8
not eat unripe mangos/fruits	20	9.1	8	17.0	4	10.3	6	6.3	2	5.3
does not know / nothing	8	3.6	5	10.6	1	2.6	2	2.1	0	
professional help*	2	0.9	1	2.1	0		1	1.0	0	
avoid contact with sick	2	0.9	1	2.1	0		1	1.0	0	
mosquito protection	1	0.5	0		0		1	1.0	0	
**Who makes your vaccine decisions?**										
oneself	151	68.6	25	53.2	23	59.0	71	74.0	32	84.2
the parents	34	15.5	10	21.3	11	28.2	10	10.4	3	7.9
the mother	9	4.1	3	6.4	1	2.6	3	3.1	2	5.3
the father	9	4.1	4	8.5	3	7.7	1	1.0	1	2.6
the partner	13	5.9	2	4.3	0		11	11.5	0	
with partner or parents	4	1.8	3	6.4	1	2.6				

* unspecific: counseling, treatment, test, except vaccination and « injection ».

URTI, upper respiratory tract infections.

LRTI, lower respiratory tract infections.

Other infections include (in descending order of frequency) tuberculosis, diarrhoea, measles, cholera, typhoid fever and AIDS.

To the question of how meningitis could be prevented, 84.5% of participants mentioned vaccination, followed by avoiding exposure to dust (14.5%, mainly by wearing nose covers or masks), and seeking professional help (7.7%, for counselling, testing and drugs other than vaccination) (**Table 2**). 2.3% mentioned avoiding eating unripe mangos and 1.4%, the application of shea butter in the nostrils. By contrast, when asked what they personally did to prevent from meningitis, only 25.9% mentioned vaccination, while half (45.9%) reported application of shea butter in the nostrils and 9.1% avoiding eating unripe mangos (**Table 2**). Vaccination was usually mentioned alone, but was sometimes combined with the application of shea butter in the nostrils, especially by older women. Avoiding dust exposure frequently was combined, especially with shea butter application in the nostrils, food hygiene and not eating unripe mangos. Surveying food hygiene, not eating unripe mangos or not knowing were more frequently mentioned by younger women (51%).

Most participants (90.6%) thought that children or “young people” (“les jeunes”) were at higher risk of meningitis, but explanations varied from higher vulnerability (rarely mentioned) to higher exposure to dust, smoke, eating unripe mangoes and poor food hygiene. Two participants mentioned gold miners and masons, due to their high dust exposure.

### Vaccination

None of the participants declared having already refused “vaccination against meningitis” (no specific vaccine was highlighted in the question). However, seven (3%) said they knew someone who had refused, due to doubts about vaccine side effects (N = 3), lack of perception of its benefit (N = 2), fear of pain (N = 1) and lack of money (n = 1). Most participants declared making their vaccine decision themselves (68.6%), while parents decide for over one third of younger women and men. For 11.5% of women aged 21–33 years, the husband was indicated as the decision maker (**Table 2**).

### Exposure to kitchen fire smoke

Only 9 (4.1%) of participants mentioned dust or smoke in respiratory tract as a meningitis risk factor and only 3 (1.9%) of participants who had mentioned dust as a risk factor for meningitis mentioned fire and kitchen smoke as the origin of such dust (**S2 Table**). Cough, sore eyes, cold or respiratory problems were mentioned each by about one third of participants as a consequence of kitchen fire smoke exposure, regardless of the age of the exposed person (babies, adults, elderly) (**S3 Table**). Most (81.2%) had heard of kitchen appliances that reduce smoke exposure, mainly gas stoves, “ROUMDE” and “Amelioré Foyer”. Most participants (95.9%) thought that children can be kept away from the smoke (by chasing them away, handing them to other people), while the lack of a person to mind the child and the child asking for their mother were mentioned as difficulties by eight participants (3.6%).

### Knowledge score

**Fig 1:**
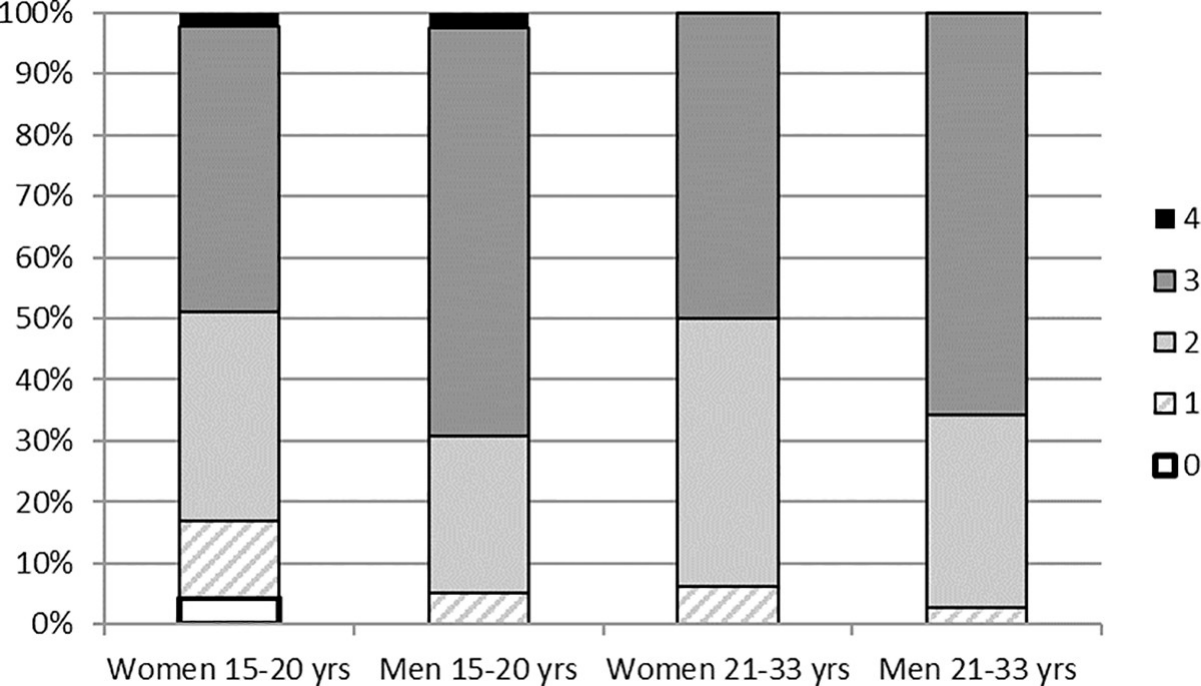
Distribution of the knowledge score of meningitis risk and prevention by age and gender among 220 young adults in Bobo-Dioulasso, Burkina Faso, 2016.

The meningitis risk and prevention knowledge scores ranged from 0 (0.9%) to 4 (0.9%), with a median of 3 (**S1 Text**). The score distribution was similar among the female (median 2) and male groups (median 3). However, younger women aged 15–20 years had higher a frequency of scores <2 (17.0%) than older women aged 21–33 years (6.3%) (**Fig 1**).

In univariate analyses, achieving at least a basic score (≥2) was associated at *P*<0.05 with having attended at least one year of junior secondary school, being able to read a newspaper and negatively associated with being a housewife or housekeeper (**S4 Table**). Univariate associations were similar for achieving a high score (≥3), showing in addition an association with male gender. In parsimonious multivariate models, age ≥21 years (OR 4.25, *P* = 0.010) and at least one year of junior secondary school (OR 14.47, *P*<0.001) both contributed significantly to having at least a basic score. Only secondary level education persisted as a determinant of a high score (OR 2.88, *P*<0.001).

In additional analyses including an age-gender term, achieving at least a basic score (≥2) tended to be substantially more likely (compared to younger women) among all other groups (e.g., older women OR 3.08, *P* = 0.050 and older men OR 7.89, *P* = 0.062) (**Table 3**). A large part of the association was explained by at least one year of junior secondary school education (M2: OR 13.09, *P*<0.001), increasing the OR for older women to 5.53 (*P* = 0.006) and decreasing the OR among males. Specifically among younger women (M3), achieving at least a basic score was strongly associated only with education (OR 23.01, *P* = 0.006).

**Table 3:** Determinants of basic (≥2) or high score (≥3) of meningitis risk and prevention knowledge among 220 young adults in Bobo-Dioulasso, Burkina Faso, 2016.

		M1	M2	M3 among women aged 15–20 yrs
		OR (*P*-value)	OR (*P*-value)	OR (*P*-value)
**Basic score (2 or more)**	Women 15–20 yrs	1	1	-
	Men 15–20 yrs	3.79 (0.105)	2.43 (0.316)	-
	Women 21–33 yrs	3.08 (0.050)	5.53 (0.006)	-
	Men 21–33 yrs	7.89 (0.062)	4.66 (0.175)	-
	At least one year of *collège*	-	13.09 (<0.0001)	23.01 (0.006)
	Sufficient information			1.74 (0.582)
**High score (3 or more)**	Women 15–20 yrs	1	1	-
	Men 15–20 yrs	2.35 (0.060)	2.03 (0.128)	-
	Women 21–33 yrs	1.04 (0.905)	1.25 (0.544)	-
	Men 21–33 yrs	2.01 (0.121)	1.69 (0.257)	
	At least one year of *collège*	-	2.50 (0.003)	5.12 (0.026)
	Sufficient information			4.48 (0.034)

OR, odds ratio.

M1: Model including only age-gender term.

M2: Model including age-gender term and junior secondary school (*college)* attendance.

M3: Model among women aged 15–20 years, including junior secondary school (*college)* attendance and satisfaction with information.

Achieving a high score (≥3) was more likely among males (e.g., younger men, OR 2.35, *P* = 0.060), but no substantial difference or trend was observed after adjusting for education, which was a significant determinant itself (OR 2.50, *P* = 0.003) (**Table 3**). Specifically among younger women (M3), achieving a high score was associated with both secondary level education (OR 5.12, *P* = 0.026) and the opinion of having sufficient information (OR 4.48, *P* = 0.034).

## Discussion

In this cross-sectional study among young adults and adolescents in Burkina Faso in 2016, 5 years after MenAfriVac® mass campaigns, we found a relatively wide range of reported knowledge, beliefs and practices of meningitis risk and prevention. Biomedically-supported facts and good practices (risk reduction in exposure to dusty dry air, vaccine prevention) were known by the majority of participants, even though a gender gap which was largely explained by secondary school attendance, persisted among young women. Local understandings and practices for risk and prevention were commonly reported and used.

Although respondents recognized the term “meningitis”, few people knew its biological etiologies, citing instead dust and dirt, often transported by wind, as well as consumption of unripe mangoes as causes. These responses were unsurprising in light of previous anthropological research in Africa and elsewhere around illness causation, and its relation to environmental and social factors, in particular malaria [15–21]. In Burkina Faso as elsewhere, lay populations may invoke biomedical categories (‘meningitis’, ‘malaria’) but explain causes of these illnesses using locally-identified etiologies [22, 23]. Dirt and dust transported by wind have been invoked in anthropological literature from this region and elsewhere as etiologies of other illnesses, and not just of meningitis. In this region with significant mango production, the availability and subsequent consumption of unripe mangoes takes place towards the end of the dry season, which coincides with the period of highest risk for meningitis and meningitis epidemics. As previously reported, unripe mango consumption is also identified by people in this region as the possible cause of other locally-recognized illnesses [22, 24].

The reported local understandings of meningitis risk and prevention must be interpreted in light of complex biomedical explanations and complex public health measures. Meningitis, defined for public health purposes as acute bacterial meningitis, has various etiologies and occurs in various age groups, but meningitis in infants usually shows unspecific clinical presentation. Meningitis vaccine prevention is complex, as it requires several vaccines (*Haemophilus influenzae* type b (Hib), pneumococci and meningococci) and vaccines are not (sufficiently) available against all pathogens (such as meningococcal serogroups C, X). These vaccines are routinely delivered only to children, although adolescents and young adults are also at risk. The link with extreme meteorological conditions is confirmed only for meningococcus and pneumococcus, and epidemics are nearly always due to meningococcus. Popular knowledge regarding the disease known under terms such as “stiff neck” therefore may mainly apply to meningococcal and pneumococcal meningitis.

Although most local preventive practices mentioned by participants are not recognized as effective, the majority actually have never been clinically evaluated. Shea butter, for instance, is widely used as a protective measure on babies’ and small children’s skin, as a barrier to prevent dirt (contamination which brings illness) from entering the bodies and making them ill [22]. For example, protecting nasopharyngeal mucosa against mechanical stress from aerosol dust and dry air may have some preventive effect, and this mechanism may be involved in the preventing invasive meningococcal disease in the African meningitis belt [4]. Locally identified risk factors should also be understood in light of incomplete biomedical interpretations of them. Although epidemiologists and public health specialists may not consider dirt and dust or mango consumption as causal factors for meningitis, participants’ explanation reflect a local logic, which underscores the confluence of multiple meteorological, air quality, and agricultural conditions: the hot dry season, high quantities of mineral aerosol dust, and the period when mango fruits are ripening. It is worth noting that biomedical understanding of these observed associations is itself incomplete, particularly the relation between increases in meningitis incidence and seasonal exposure to Harmattan winds [1].

The link between smoke exposure and pneumonia is well established [25, 26], and participants frequently mentioned respiratory problems due to fire smoke exposure. Childhood smoke exposure remains a particular problem, especially in the absence of a second caregiver. The lack of knowledge about the link between smoke exposure and meningitis can be explained by the fact that public health communication does not include this association, as only two studies provide evidence for meningitis risk during epidemics [7, 8] and one for meningococcal carriage [27].

Study participants rarely mentioned interpersonal disease transmission, despite its etiological importance for an infectious disease such as meningitis. In Hayden et al. [13] only half of the participants mentioned crowding as a risk factor and one-third isolating sick persons as preventive measures against meningitis. It is possible that the fact that the disease is transmitted from and to family and community members is not socially acceptable and thus views were held back during the interview. In West Africa and Burkina Faso, other illnesses can have important social dimensions, usually related to the manipulation of occult forces, but because of the sensitivity of this concern, it remains unlikely that study participants would raise these social concerns in this type of study [15, 18, 21, 28–30]. The meaning of physical distancing and appropriate ways to promote it during epidemics, including the current Covid-19 pandemic, may be worth investigating further.

The educational gender gap has been targeted by organizations such as UNICEF over decades. Less is known about a gender gap for health literacy in sub-Saharan Africa, in particular infectious disease prevention apart from sexually transmittable diseases. One study in Ghana recently described a similar gender gap, in part related to educational differences, and suggested that “dominant male gender roles and differing expectations and opportunities for socio-economic advancement for men and women” may lead to the observed differences [31]. Closing this gender gap would be important not only for women, but also to ensure that families make the best health care decisions for their children.

The additional difference between women below and above age 21, which persisted after adjustment for education, can be interpreted in at least two ways: firstly, women aged ≥21 years were old enough in 2010 to understand and retain the information provided during the MenAfriVac communication campaign, whereas the younger age group (<15 years in 2011) did not benefit from this information campaign even if they were vaccinated. This interpretation would suggest that such campaigns have long-term benefits for knowledge transmission and retention and should be repeated periodically to catch-up with younger cohorts. A second interpretation is that women may receive health information primarily from health professionals during perinatal care, while men receive it through various other channels. Women under 21 years of age in this urban population would have been less exposed to educational information from health professionals, in the absence of an early pregnancy. This interpretation implies that for infectious diseases, including sexually transmittable diseases and human papilloma virus, where infection can occur before young women and adolescents have consistent access to health care professionals, specific educational interventions are needed outside school and health care facilities.

More than half of study participants—and two thirds of young women—claimed that they did not receive sufficient information about meningitis prevention; most expected this information to come from health professionals and the media. There is a crucial role to be played by public health authorities, health workers and the media in improving health literacy and promoting vaccine uptake. Such informational transfers can take place in many forms, from exchanges at health centers to radio announcements, but should, as pointed out above, include settings frequented by young women who are no longer at school and not yet seen in perinatal care. Interventions should be consistent and respectful of local explanations and logics around meningitis risk and prevention. Eighty-two percent of respondents mentioned vaccination as a means of prevention, which suggests that public health messaging around meningitis prevention does reach populations. Participants rarely reported vaccine refusal by themselves or people they know; when they did, participants reported reasons associated with vaccine hesitancy as defined by WHO [32]: concerns about vaccine safety (confidence), doubts about vaccine effectiveness (complacency), and lack of money (convenience). Our findings echo previous anthropological studies among Burkina Faso populations, which have pinpointed the coexistence of biomedical and local practices and have found limited levels of understanding of what vaccines ‘do’, how they work and how they are produced, in parallel to high acceptance of vaccination in general [21, 33, 34]. Such insights confirm earlier anthropological findings that people do not need to know a vaccine’s contents, actions, or targeted diseases in order to accept it [35]. Previous anthropological investigations in Burkina Faso have found some level of questioning among informants about the effectiveness of vaccines: both the pentavalent infant vaccination and vaccines in general [21, 36]. Populations living in the meningitis belt generally accept meningococcal vaccines, but there is a risk that hesitancy may increase once epidemics recede as a consequence of successful preventive vaccination programs.

### Limitations

Our study has several limitations. By nature, information was self-reported and may be subject to social desirability bias, as participants may have perceived pressure from the interviewing nurse or from their social environment regarding expected beliefs. We sought to reduce this bias by investigating participants’ and their networks’ perceptions and practices: What do others think? What do you think? What do you do?

Our results are limited to an urban and peri-urban population and cannot be extrapolated to a rural population, where the balance between access to information and the importance of local beliefs may be different. Ethnic specificities may have influenced our results; however, Bobo-Dioulasso is an economically significant town and its inhabitants come from various regions of Burkina Faso and West Africa for trade and higher studies, such that it is unlikely that our results are dominated by one single ethnicity.

Our definition of the knowledge score was based on a binary categorization of responses as “correct” and “incorrect” and it included some questions to which even health care workers may have found difficult to reply to correctly. In addition, for some questions, several responses were possible. A person could get one point for giving an expected response, while also citing other incorrect aspects. Finally, the sample size was not sufficient for some stratifying analyses on gender, age and socioeconomic status.

At the same time, however, our methodology, which combines a knowledge, attitude, beliefs and practice (KABP) study with a qualitative component, demonstrates the usefulness of this type of mixed approach. KABP studies have been roundly criticized for putting complex perceptions into simplistic boxes, but remain in widespread use. By adding qualitative, open-ended questions, we were able to gain access to rich explanatory data on practices and understandings related to risk and prevention of meningitis that we otherwise would not have collected.

## Conclusion

In conclusion, our study shows a gender gap in knowledge about meningitis risk and prevention, largely due to education inequalities in Burkina Faso. Women aged 20 or younger had particularly low levels of knowledge and should be targeted by interventions outside schools and perinatal care. Our study suggests a strong adherence among young adults to local understandings of and practices around meningitis risk and prevention. National and international vaccination initiatives need to take these local understandings and practices into account.

## Supporting information

S1 Text Knowledge score development and distribution. (DOC)

S1 Table Knowledge, beliefs and practice questions. Original in French and authors’ translation into English.
(PDF)

S2 Table Themes mentioned by participants on specific questions about meningitis knowledge, beliefs and practice. Participants could provide more than one theme.
(PDF)

S3 Table Themes mentioned by participants on effects of kitchen fire smoke exposure. Participants could provide more than one theme.
(PDF)

S4 Table Association of participant characteristics and knowledge score ≥2 and ≥3. Estimates were obtained from bivariate logistic regression models.
(PDF)

S1 Dataset (DTA)
