# Supplementary material for: Knowledge, beliefs and practices regarding prevention of bacterial meningitis in Burkina Faso, 5 years after MenAfriVac mass campaigns
Source: PLoS One. 2021 Jul 14;16(7):e0253263. doi: 10.1371/journal.pone.0253263 (PMC8279338; doi:10.1371/journal.pone.0253263)
Supplement: S1 Text — (DOC) [file pone.0253263.s001.doc]

**S1 Text. Knowledge score development and distribution**

**Table A in S2 Text. Development of knowledge score: biomedically correct responses in the perspective of meningitis prevention.** Questions were open.

| **Questions** | **Biomedically correct responses in the perspective of meningitis prevention** |
| --- | --- |
| What is the effect of dry air on health during the dry season? | Meningitis |
| In your opinion, are there people who are more at risk of getting the disease? | Children (and young people) |
| In your opinion, how does one contract meningitis? | Transmission from person to person |
| In your opinion, how should one prevent meningitis? | Vaccination |

Table B in S2 Text. Meningitis knowledge score distribution among participants

| Knowledge score | Distribution, N (%) |
| --- | --- |
| 0 | 2 (0.9) |
| 1 | 15 (6.8) |
| 2 | 80 (36.4) |
| 3 | 121 (55.0) |
| 4 | 2 (0.9) |
|  | 220 |
