# Supplementary material for: Knowledge, beliefs and practices regarding prevention of bacterial meningitis in Burkina Faso, 5 years after MenAfriVac mass campaigns
Source: PLoS One. 2021 Jul 14;16(7):e0253263. doi: 10.1371/journal.pone.0253263 (PMC8279338; doi:10.1371/journal.pone.0253263)
Supplement: S1 Table — Original in French and authors’ translation into English. (PDF) [file pone.0253263.s002.pdf]

S1 Table. Knowledge, beliefs and practice questions. Original in French and authors' translation into English

Authors' translation from French into English.

Topic	Subtopic	Question	Original question in French
Meningitis prevention	Knowledge about risk factors	In your surroundings, how do people think meningitis is got?	Dans votre entourage, comment les gens pensent qu'on attrape la méningite ?
		In your opinion, how does one get meningitis?	Selon vous, comment peut-on attraper la méningite ?
		In your opinion, are there people who are more at risk of getting meningitis?	Selon vous, est-ce qu'il y a des personnes qui sont plus à risque d'attraper la méningite ? Qui et pourquoi ?
		If dust or dirt is mentioned: where does dust/ dirt and pollution come from?	[*S'il y a mention de la saleté, pollution, poussières etc.*] D'où vient la saleté/la pollution/la poussière ? (adapter à la notion évoqué par le participant)
	Preventative knowledge and practice	In your opinion, how can one prevent meningitis?	Selon vous, comment peut-on prévenir la méningite ? (plusieurs réponses possibles ; relatif à la médecine traditionnelle ou la médecine moderne)
		What do you do to protect yourself from meningitis?	Pour vous, qu'est-ce que vous faites pour prévenir la méningite ?
	Accessibility of information	Do you think the information concerning protection against meningitis is sufficiently accessible to you? Where do you get it from?	Pensez-vous avoir suffisamment accès à l'information concernant la protection contre la méningite ? De qui ?
Vaccination	Refusal	Would you refuse vaccination against meningitis? For which reason?	Vous personnellement, refuseriez-vous la vaccination contre la méningite ? Pour quelle raison ?
		Someone in your surrounding ever refused meningitis vaccination? Which were the reasons?	Quelqu'un dans votre entourage a-t'il refusé la vaccination contre la méningite ? Pour quelle raison ?

	Decision making	Who helps you in vaccination decision making?	Qui vous aide à prendre la décision relative à la vaccination contre la méningite ?
Effect of dry air	Knowledge about health effects	What is the effect of dry air?	Pour vous personnellement, qu'est-ce qui est l'effet sur la santé ou le bien-être de l'air sec pendant la saison sèche ?
	Preventative practice	What do you do to protect from these effects of dry air?	Qu'est-ce que vous faites pour alléger cet effet ? (si l'effet est désagréable ou perçu comme dangereux)
Kitchen fire smoke exposure	Practice of smoke exposure	Location of kitchen<br>Fuel used for cooking<br>If children are exposed to kitchen smoke, it is possible to remove them from smoke exposure? How?	La cuisine de votre ménage est située<br>dans une pièce fermée ☐ sous un hangar ☐ en plein air ☐<br><br>Combustible le plus couramment utilisé : bois ☐ charbon ☐ gaz ☐ autres (préciser) ☐<br><br>Dans les ménages où les enfants sont exposés à la fumer : serait-il possible d'écarter les enfants de l'exposition à la fumée de cuisine ou chauffage ?<br><br>Si OUI, comment ?<br>Si NON, quelles sont les difficultés attendues ?
	Knowledge about prevention against smoke exposure	Have you heard of kitchen appliances that reduce exposure to smoke? Which?	Avez-vous entendu d'équipements de cuisine qui permettent de réduire l'exposition aux fumées ?<br>Oui □ Précisez lesquels :<br>Non □<br><br>En utilisez-vous un ? Oui ☐ Non ☐<br>Raison pourquoi non :

	In your opinion, what health effects can have a longer exposure to smoke (in kitchen, from heating). Please name the type of effect, whether it is good or bad and which age are concerned.<br><br>On babies:<br>On adults<br>On the elderly	Selon vous, quels effets sur la santé peut avoir l'exposition prolongée à la fumée (de cuisine, chauffage…)? Précisez le type de chaque effet, si c'est bon ou mauvais, et les âges concernés<br><br>-Chez le Bébé :<br>-Chez l'adulte :<br>-Chez la personne âgée :
