# Supplementary material for: Knowledge, beliefs and practices regarding prevention of bacterial meningitis in Burkina Faso, 5 years after MenAfriVac mass campaigns
Source: PLoS One. 2021 Jul 14;16(7):e0253263. doi: 10.1371/journal.pone.0253263 (PMC8279338; doi:10.1371/journal.pone.0253263)
Supplement: S2 Table — Participants could provide more than one theme. (PDF) [file pone.0253263.s003.pdf]

S2 Table. Themes mentioned by participants on specific questions about meningitis knowledge, beliefs and practice. Participants could contribute more than one theme.

**In your environment, how do people think meningitis is contracted?**	N (%)
Unripe mangos/fruits	110 (50.0)
Dust	107 (48.6)
Does not know / has not heard from people	20 (9.1)
Poor food hygiene, dirt	17 (7.7)
Wind	12 (5.5)
Dust or smoke in respiratory tract	9 (4.1)
Sun	3 (1.4)
Person-to-person	2 (1.0)
**Origin of dust, if dust was mentioned in personal opinion on risk factors** (N=155)	
Wind	115 (74.2)
Inhaled dust	35 (22.6)
Unhygienic conditions	4 (2.6)
Fire and kitchen smoke	3 (1.9)
Not specified dust	2 (1.3)
Traffic pollution	2 (1.3)
**In your opinion, are there people more at risk of getting the disease?**	
Children (reasons: vulnerable, dust- and smoke exposure, unripe fruit, low food hygiene)	200 (90.9)
Young people (reasons: much outside)	9 (4.1)
Everyone	9 (4.1)
Does not know	3 (1.4)
Goldminers, masons (dust exposure)	2 (1.0)
