# Supplementary material for: Knowledge, beliefs and practices regarding prevention of bacterial meningitis in Burkina Faso, 5 years after MenAfriVac mass campaigns
Source: PLoS One. 2021 Jul 14;16(7):e0253263. doi: 10.1371/journal.pone.0253263 (PMC8279338; doi:10.1371/journal.pone.0253263)
Supplement: S3 Table — Participants could provide more than one theme. (PDF) [file pone.0253263.s004.pdf]

S3 Table. Themes on effects of kitchen fire smoke exposure mentioned by participants. Participants could contribute more than one theme.

	N (%)
**Kitchen fire smoke exposure effect of smoke exposed to babies**	
« cough »	80 (35.9)
« sore eyes »	54 (24.2)
« cold »	82 (36.8)
« respiratory problems »	68 (30.5)
«meningitis »	1 (0.5)
**Effect of smoke exposed to adults**	
« cough »	72 (32.3)
« sore eyes »	71 (31.8)
« cold »	78 (35.0)
« respiratory problems »	73 (32.7)
**Effect of smoke exposed to the elderly**	
« cough»	69 (30.9)
« sore eyes »	52 (23.3)
« cold »	62 (27.8)
« respiratory problems »	75 (33.6)
**Have you ever heard of kitchen appliances that reduce smoke exposure? Which.**	
Yes	181 (82.3)
Gas stove (« Gasherd »)	81 (36.8)
« ROUMDE »	62 (28.2)
«Amelio Foyer»	60 (27.2)
Charcoal	23 (10.5)
**If children are exposed, is it possible to remove them from smoke exposure?**	
Yes	211 (95.9)

S5 Table. Association of participant characteristics and knowledge score ≥2 and ≥3. Estimates were obtained from bivariate logistic regression models.

		Score ≥2		Score ≥3	
		**OR**	**P-value**	**OR**	**P-value**
Age (years)	15-20 yrs	1		1	
	21-33 yrs	2.39	0.090	0.86	0.594
Gender	Female	1		1	
	Male	2.68	0.131	2.11	0.011
Vaccinated with MenAfriVac	No	1		1	
	Document-confirmed	-*		1.34	0.474
	Recall	2.18	0.124	1.57	0.112
Highest level of education	No schooling or Lower primary (*CP*)	1		1	
	Upper primary (*CM2*)	1.78	0.331	0.78	0.609
	Junior secondary school (*collège*)	8.52	0.003	1.77	0.183
	Senior Secondary school (*Lycée et Bac+*)	-*		4.22	0.003
At least one year of junior secondary		10.14	<0.001	2.79	<0.001
Can read a newspaper	No	1		1	
	A bit	2.89	0.339	0.73	0.622
	yes	5.41	0.003	2.13	0.029
Number of people who share a meal	1-6	1		1	
	7-9	0.85	0.791	1.21	0.571
	10-47	1.12	0.856	1.73	0.089
Situation of kitchen	Enclosed	1		1	
	Hangar	- *		1.23	0.747
	Open air	1.44	0.475	0.58	0.053
Profession	Student	1		1	

	Housewife, housekeeper	0.14	0.005	0.41	0.012
	Vending	1.03	0.980	0.52	0.094
	Artisan	0.21	0.068	0.76	0.560
	Employee	0.18	0.165	0.73	0.687
	Civil servant	- *		- *	
Believes having sufficient information on meningitis prevention		2.88	<0.001	2.01	0.012

* not estimated due to empty cells
