# Supplementary material for: Knowledge, beliefs and practices regarding prevention of bacterial meningitis in Burkina Faso, 5 years after MenAfriVac mass campaigns
Source: PLoS One. 2021 Jul 14;16(7):e0253263. doi: 10.1371/journal.pone.0253263 (PMC8279338; doi:10.1371/journal.pone.0253263)
Supplement: S4 Table — Estimates were obtained from bivariate logistic regression models. (PDF) [file pone.0253263.s005.pdf]

S4 Table. Association of participant characteristics and knowledge score ≥2 and ≥3. Estimates were obtained from bivariate logistic regression models.

		Score ≥2		Score ≥3	
		**OR**	**P-value**	**OR**	**P-value**
Age (years)	15-20 yrs	1		1	
	21-33 yrs	2.39	0.090	0.86	0.594
Gender	Female	1		1	
	Male	2.68	0.131	2.11	0.011
Vaccinated with MenAfriVac	No	1		1	
	Document-confirmed	-*		1.34	0.474
	Recall	2.18	0.124	1.57	0.112
Highest level of education	No schooling or Lower primary (*CP*)	1		1	
	Upper primary (*CM2*)	1.78	0.331	0.78	0.609
	Junior secondary school (*collège*)	8.52	0.003	1.77	0.183
	Senior Secondary school (*Lycée et Bac+*)	-*		4.22	0.003
At least one year of junior secondary		10.14	<0.001	2.79	<0.001
Can read a newspaper	No	1		1	
	A bit	2.89	0.339	0.73	0.622
	yes	5.41	0.003	2.13	0.029
Number of people who share a meal	1-6	1		1	
	7-9	0.85	0.791	1.21	0.571
	10-47	1.12	0.856	1.73	0.089
Situation of kitchen	Enclosed	1		1	
	Hangar	- *		1.23	0.747
	Open air	1.44	0.475	0.58	0.053
Profession	Student	1		1	

	Housewife, housekeeper	0.14	0.005	0.41	0.012
	Vending	1.03	0.980	0.52	0.094
	Artisan	0.21	0.068	0.76	0.560
	Employee	0.18	0.165	0.73	0.687
	Civil servant	- *		- *	
Believes having sufficient information on meningitis prevention		2.88	<0.001	2.01	0.012

* not estimated due to empty cells
